# Bridging communities, prevention, and heart health: U.S. strategies for CHW cardiovascular training and integration

**DOI:** 10.3389/fepid.2025.1597970

**Published:** 2025-12-16

**Authors:** Akua G. Asare, Melvin R. Echols

**Affiliations:** 1 American College of Cardiology, Washington, DC, NW, United States; 2 Department of Medicine, Morehouse School of Medicine, Atlanta, GA, United States

**Keywords:** community health workers, cardiovascular health, health equity, medication adherence, culturally competent care, faith-based health initiatives, insurance navigation, chronic disease management

## Abstract

**Background:**

In the United States, cardiovascular disease (CVD) disproportionately affects communities facing adverse social determinants of health (SDOH). Community Health Workers (CHWs) can bridge gaps in trust, navigation, and culturally tailored education.

**Methods:**

We conducted a U.S.–focused narrative review (2015–2025) of PubMed, Scopus, and Google Scholar, prioritizing empirical evaluations of CHW-led CVD interventions, training models, integration strategies, and financing mechanisms. International CHW programs were used only to extract practices transferable to U.S. delivery and payment contexts.

**Results:**

Multidisciplinary team-based care demonstrates that engaging CHWs in US regions improves blood pressure control and medication adherence. Economic evaluations increasingly support CHW models for CVD prevention and control. Effective programs specify CHW task bundles (e.g., self-measured BP onboarding, adherence coaching, care navigation, SDOH linkage) and align training with national core competencies. Integration pathways include clinic-embedded, payer-based, public health, and community-based partnerships. U.S. reimbursement options are emerging through Medicare Community Health Integration/Principal Illness Navigation and state Medicaid mechanisms. Faith-based collaborations can extend reach when coupled with standardized training and simple outcome tracking.

**Conclusions:**

For U.S. health systems and payers, immediate priorities are (1) competency-based CHW training with cardiac modules, (2) sustainable reimbursement tied to cardiovascular quality metrics, and (3) a minimal outcome set to demonstrate value. Global best practices should be adapted to the U.S. scope-of-practice, supervision, and documentation requirements to scale equitable CVD care.

## Introduction

Cardiovascular disease (CVD) is the leading cause of mortality worldwide. It is disproportionately driven by the social determinants of health (SDOH), conditions in which people are born, grow, work, live, and age (1). For underserved and certain racial and ethnic groups, institutional and systemic policies intensify the SDOH, leading to inequities in access to preventative or chronic care and available treatments (2).

Community Health Workers (CHWs) have been recognized as vital for providing healthcare to underserved populations in the United States for over 70 years (3). However, there are many international models of CHWs. The American Public Health Association (APHA) defines a CHW as a “frontline public health worker who is a trusted member of an organization or has an unusually close understanding of the communities they serve.” (4) As this APHA definition has been used in the US to codify a lawful mandate for the provision of care, many states have adopted and continue to use it to define the practical scope of CHWs. However, despite growing evidence to support CHW-led interventions, inconsistent training and certification, limited reimbursement models, and fragmented coordination with healthcare systems threaten the optimization of CHW programs (5). This perspective aims to evaluate the impact of CHWs on cardiovascular health outcomes in the United States (U.S.), while assessing global evidence-based models of training, education, and integration into healthcare systems to maximize patient benefits.

**Figure 1:**
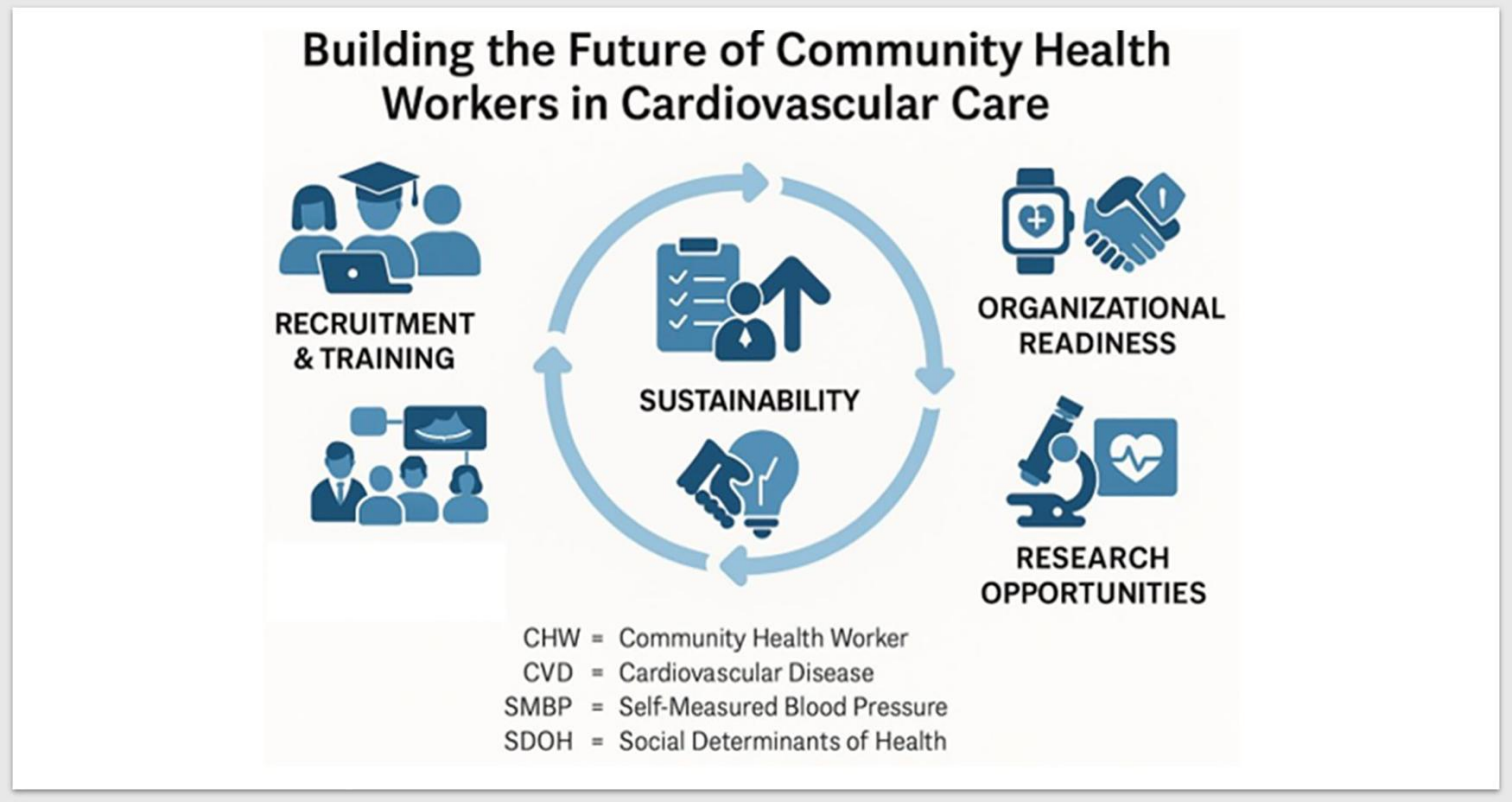
Central illustration of potential U.S. Community Health Worker Capacity.

This review targets CVD inequities in the U.S., particularly by synthesizing delivery and payment evidence to incorporate best practices from mature international CHW programs, where applicable. Transferability is assessed by alignment with U.S. CHW roles and supervision, feasibility under U.S. credentialing and scope-of-practice requirements, and relevance to CVD quality metrics (see Figure 1). Thus, our review provides a U.S.-specific roadmap for CHW training and care team integration, payment, and other processes informed by global practices.

## Methods

We performed a narrative review (January 2015–October 2025) of PubMed, Scopus, and Google Scholar. Because the aim is U.S. implementation, we prioritized U.S. studies and policy documents. We included non-U.S. evidence only when CHW tasks aligned with U.S. core competencies and could be delivered under American supervision, documentation, and reimbursement requirements. Two reviewers screened titles/abstracts, resolved disagreements by discussion, and extracted setting, CHW role, training, integration model, outcomes (clinical, utilization, cost), and financing features. We synthesized findings thematically across effectiveness, workforce/training, and payment. Limitations include selection and publication bias, and heterogeneity precluding meta-analysis.

We used iterative keyword combinations for cardiovascular care, community health workers, team-based care, reimbursement, and training models. We prioritized U.S.-based studies and included international models only when CHW tasks aligned with U.S. core competencies and supervision requirements. We excluded volunteer-only models and non-health CHW programs. Two reviewers screened titles and abstracts independently, resolved disagreements by discussion, and extracted data on setting, CHW role, training, integration, clinical outcomes, utilization, and financing. Transferability was assessed using U.S. documentation requirements and realistic reimbursement options (Medicare CHI/PIN; Medicaid SPAs, 1115 waivers, and MCO contracts).We focus specifically on CVD because hypertension, dyslipidemia, heart failure, and stroke exhibit some of the most persistent inequities in the U.S. among race, ethnicities, and geographic regions. Consequently, CHWs have demonstrated measurable effects on these cardiometabolic pathways.

## Results

### CHW impact on cardiovascular health

Across U.S. randomized trials and program evaluations, CHW-enabled team-based care consistently improves blood pressure control, medication adherence, and cardiometabolic risk among underserved populations (1–5). Findings prioritize U.S. evidence, with international examples flagged and paired with brief ‘U.S. translation notes’ to indicate how the practice would operate within American delivery and payment systems. Based on the evaluated studies, the evidence supports the use of CHW-led interventions, demonstrating improved outcomes across several underserved populations and communities with challenges accessing healthcare (6–8). The trusting relationship described enables CHWs to serve as intermediaries between health and social services, facilitating access to quality services through culturally tailored education (7, 9, 10). Across randomized and program evaluations, CHW-enabled team-based care improves blood pressure control and adherence, particularly in immigrant and underserved populations (11–14). In some regions of the world, CHW-led interventions have been associated with shorter hospital stays and lower mortality rates (1, 6, 15, 16). CHW-led interventions can be effective in both mental health and chronic-care models. Recent evidence also suggests that, for patients with comorbid cardiovascular and mental health conditions, CHW-delivered interventions can lead to greater medication adherence and improved quality of life (17). In addition to other conditions, the greater use of primary care services, secondary to more straightforward healthcare system navigation, has been associated with navigation-focused CHW interventions for patients with CVD (2, 10, 18). The Community Preventive Services Task Force (CPSTF) supports CHW-integrated team-based care models to improve education-based outreach and address potentially modifiable risks (19).

CHW-led interventions for screening and chronic illnesses, such as HIV, also enhance screening and treatment rates compared to standard care practices (10, 20–23). They guide individuals navigating complex health systems and collaborate with community members and health and social service entities to increase adherence and promote appropriate service use. Studies also indicate substantial cost advantages in cardiovascular disease prevention for specific populations, including HIV patients, potentially decreasing ischemic heart disease, strokes, and cardiovascular disease mortality by 9%–36% in West Africa (24).

A recent study in Rwanda demonstrated the effectiveness of CHW-led use of a body mass index risk tool in assessing CHWs’ ability to screen and identify community members at risk of CVD. These findings suggest a positive association between CHW-conducted risk assessments and those performed by nurses, indicating that CHWs can be effective and reliable sources to screen for CVD in high-risk communities (25). A secondary analysis of two patient-centered lifestyle interventions that involved CHWs among South Asians in New York City revealed a significant reduction in blood pressure among patients with both diabetes and hypertension. These interventions included a culturally adapted diabetes management initiative, the DREAM project, and a hypertension management program, Project IMPACT (14).

### Economic evidence

Economic evaluations increasingly support CHW models for CVD. In Colorado's statewide CHW program, a cost-effectiveness analysis found that the intervention was cost-effective compared to standard approaches, driven by reductions in risk factors achieved in community settings (26). These data align with the CPSTF's economic findings for team-based blood pressure care and inform scale-up through Medicare Community Health Integration (CHI) services (HCPCS codes G0019/G0022) and state Medicaid financing pathways (SPAs, 1115 waivers, MCO contracts) (27–29).

### Challenges in CHW implementation

When recruiting CHWs from within a community without a specific order, several studies help identify and engage community members at the volunteer or paid work level (7, 21). Some of these methods directly focus on specific vulnerable populations through a partnership-based model of care (22, 30–32). For many middle- and low-income countries, CHW interventions often result from volunteer efforts. However, incentivizing CHWs with pay or other benefits is associated with better learning and overall performance (33–35). Therefore, recruitment efforts should focus on individuals from the target populations they will serve, preferably developed in partnership with community members with strong advocacy voices (3, 36). This process should involve assessing their understanding of community morals, ethics, perspectives, dynamics, concerns, and resources.

An organization should establish clear selection and commitment criteria, effective retention strategies, and adequate compensation when recruiting CHWs within communities. Networking with CHW associations, local health departments, non-profits, and community-based organizations, and faith-based organizations (FBOs) can enhance the benefits of CHW intervention and often expedite gaining trust at the patient level. Collaboration with community stakeholders is, therefore, essential. Health systems can utilize a variety of communication platforms, such as social media and radio, to raise awareness.

The screening capabilities of a well-trained CHW are also crucial in identifying patients with the highest potential for success. However, to demonstrate effectiveness in reducing cardiovascular disease or modifiable risk factors, CHW-led interventions will require effective training methods for the continual need to address cultural beliefs or historical stigmas related to CVD. Ultimately, effectiveness should be characterized with outcome measures that adequately assess the intervention.

### Training and standardization challenges in the US

While most US states have certification and training programs, the lack of a standardized national training approach for CHWs becomes problematic when considering the scalability of various programs. Several entities provide CHW training, including local and state CHW organizations, public health departments, educational institutions, and Care-Based Organizations (CBOs). As of May 2024, 11 states—Arizona, Connecticut, Colorado, Kentucky, Massachusetts, Maryland, New Mexico, Ohio, Oregon, Utah, and Texas—had established credentialing and certification programs through their state public health departments. Six states (Florida, Missouri, Pennsylvania, Rhode Island, and Virginia) offer private programs led by independent credentialing boards, while six others (Arkansas, Indiana, Kansas, North Carolina, South Carolina, and South Dakota) offer programs led by CHW associations or individual CHWs (37). Five states (California, North Dakota, Illinois, Tennessee, and Mississippi) are also developing their programs. Some states, such as Louisiana, have focused on creating more comprehensive training programs rather than establishing credentialing and certification programs. While licensure is not required in many states, some require it for service reimbursement (37).

According to the National C3 Council, CHWs should possess 11 core skills: communication; interpersonal and relationship building; service coordination and navigation; capacity building; advocacy; enhancing individual and community capacity; personal and community assessment; outreach; professional skills and conduct; evaluation and research; and knowledge base (38). As communities have varying needs, CHW curricula should cover general and community-specific topics and use multiple modes of adult learning. This includes hands-on training, interactive role-playing sessions, team-based exercises, and various in-the-field resources, such as worksheets, modules, manuals, as well as in-field shadowing and internship opportunities (23, 39–41).

Training should also include information on local and state-specific regulations for certification and credentialing, as well as education on navigating the healthcare system and collaborating with healthcare professionals. Additionally, it should provide instruction on duty supervision, mentoring, and coaching (16, 36, 39, 42, 43). However, future research should focus on identifying the specific activities and core competencies that can be universally integrated into specialized CHW training programs, enabling regional flexibility.

### Integration into cardiovascular U.S. healthcare and community settings

**Figure 2:**
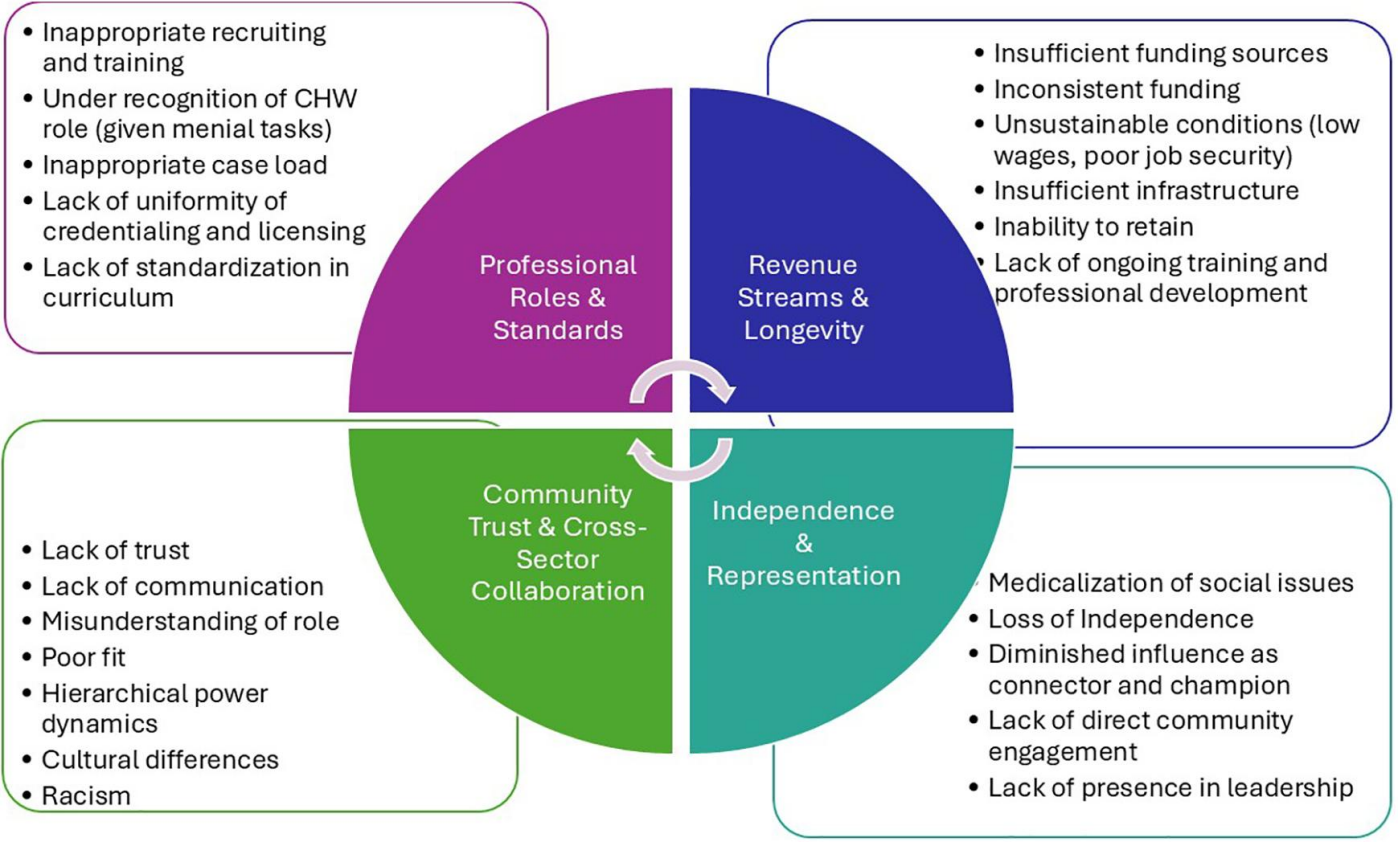
Challenges to integration of CHW into healthcare system models.

CHWs can work for a variety of organizations, including healthcare organizations, public health departments, nonprofits, social service agencies, educational institutions, insurance providers, corporations, and faith-based groups (see Table 1). Approximately 80% are employed by nonprofit organizations, community clinics, and hospitals (7, 44). They are integrated into healthcare systems in four main ways: through community-based partnerships with clinics, team-based care in hospitals, payer-based models, and public health departments (3, 6, 7, 45). Integrating trained CHWs into the community for intervention purposes is often hindered by several factors, some of which are unique to each community. In a community-based partnership model, CHWs focus on outreach and referrals (Figure 2).

**Table 1:** Community health worker cardiovascular task bundles, core competencies, documentation, primary outcomes, and U.S. Payment Routes.

CHW task bundle	Core competency/required training	Required documentation	Primary outcomes	U.S. payment source
SMBP training/coaching	Communication; assessment; education; escalation protocol	Time spent, device education, reading reviews, escalation if thresholds met	SBP/DBP control; reduced ED visits	Medicare CHI monthly; Medicaid SPA/MCO; VBP attribution
Adherence and refill engagement	Relationship-building, behavior change, navigation	Barriers identified, plan; follow-up	↑PDC: fewer gaps in therapy	Medicare CHI/PIN; Medicaid SPA/MCO; ACO care-management
Care navigation support	Service coordination; systems navigation	Appointment arranged; barriers resolved	↑Primary-care follow- up; ↓no-show rate	CHI/PIN; Medicaid case-management; VBP
SDOH linkage (food/housing/utilities)	Resource mapping; advocacy	Need documented; referral completed; confirmation of service	↑Access to resources; ↓avoidable utilization	CHI + SDOH assessment; Medicaid instead of services (state-dependent)
Post-discharge transitions	Coordination; communication; coaching	Contact within 48–72 h; meds reviewed; red-flags addressed	↓30-day ED/admits	CHI/PIN; Medicaid case-management; hospital VBP
Group education in CBOs/FBOs	Education; facilitation; cultural tailoring	Session roster, topics, goals set	↑Health literacy; ↑self-management	Grant/contract; Medicaid health-related services; VBP community partnership

ACO, accountable care organization; CM, care management; CHI, community health integration; PIN, principal illness navigation; SPA, (Medicaid) state plan amendment; MCO, managed care organization; VBP, value-based payment; SMBP, self-measured blood pressure; SBP, systolic blood pressure; DBP, diastolic blood pressure; PDC, proportion of days covered; SDOH, social determinants of health; ED, emergency department; EHR, electronic health record; ILOS, in-lieu-of services; QHP, qualified health professional.

In team-based care, CHWs collaborate with other healthcare providers within a hospital, clinic, or facility, offering valuable insights into the barriers patients face in accessing care. Each setting requires a focused skill set or competency that CHWs must acquire. In payer-based models, insurance companies and state Medicare/Medicaid agencies utilize CHWs to support patients within their respective networks. The CHWs trained in this capacity must have a certain level of numeracy to guide patients. Therefore, a well-trained CHW for complex tasks may require education beyond high school. Similarly, public health departments engage community health CHWs to provide services to healthcare providers and entities. Consequently, strategies for successfully integrating CHWs into healthcare teams vary widely. However, this approach must focus on the maximal benefits of CHW interventions.

The growing demand for the expedient integration of telemedicine and artificial intelligence (AI)-enabled tools has presented both opportunities and challenges. On one hand, training CHWs to utilize telehealth platforms, wearable devices, and AI-driven tools could revolutionize our concept of ambulatory medicine. Particularly for patients with CVD and other chronic conditions, the innovative use of CHWs in this sense would likely yield effectiveness and sustainability. In contrast, many communities with a higher SDOH burden also have limited broadband access, hindering community digital literacy, particularly among veterans, older members, and school systems (46, 47). Healthcare teams can also empower CHW-led programs by identifying team champions who can advocate for CHWs and their work. This advocacy includes ensuring that team members receive formal education on the unique roles and characteristics of CHWs. Additionally, standardized guides should be developed to help CHWs understand the healthcare system, including practitioners’ roles and responsibilities. As in any associated research, incorporating community health workers (CHWs) into the program's ongoing development and evaluation is crucial. Continuous professional and technical development for CHWs is essential, as is support in creating patient education materials. Professional development should emphasize core competencies, such as cultural sensitivity, updates in health education, practical role-playing skills, and ongoing supervision and observation by supervisors (3, 33).

## Discussion

Although we reference international CHW programs to illustrate best practices, our implementation and financing guidance is tailored to U.S. delivery systems. Where we cite non-U.S. evidence, we provide a U.S. translation that describes the required supervision, documentation, and payment pathways. CHW-led interventions for CVD management are promising. However, several challenges remain for current and future programs. CHW work continues to have inconsistencies in incentives or pay for work, which decreases the sustainability of engagement and the feasibility of pilot initiatives. Depending on the level of funding support, there may be a directed focus on a particular treatment pathway, which can limit the comprehensive use of training in a well-developed CHW curriculum. CHWs working with insurance companies often find themselves limited to making referrals, rather than engaging directly with the community (42, 48). The level of engagement from public health departments with CHWs can vary significantly. Thus, CHWs must maintain continuing education to retain a high level of training proficiency.

Faith-based organizations serve large numbers of individuals at high cardiovascular risk and maintain strong community trust, positioning them as natural partners for CHW-led CVD initiatives. Their established communication channels and social support networks can be leveraged to deliver structured hypertension coaching, lifestyle programming, SMBP training, and medication adherence support. Faith and religion are crucial sources of encouragement, strength, and social support for many individuals. FBOs are charitable or nonprofit entities associated with religious groups or belief systems. These organizations provide spiritual guidance and are often trusted community providers of social services and resources. Their emphasis on moral and ethical beliefs, values, practices, and religious authority drives community-focused initiatives, interventions, and programs. With deep roots in their communities, FBOs are well-positioned to promote the well- being of their congregations and the broader community. Consequently, they are particularly suitable for providing health-based programs (49). For many chronic conditions, religious and spiritual practices have been recognized as effective coping strategies.

Faith-based health promotion is a foundational means of galvanization in American healthcare. Historically, FBOs have played a vital role in addressing local and national health emergencies and disasters, promoting health awareness, combating vaccine hesitancy, and managing stress (50–54). FBOs have also been instrumental in promoting healthy lifestyle changes, such as nutrition, exercise, and the psychological aspects of discipline. Thus, CHW-led intervention in these spaces can perform quite well (51). The lifestyle-modifying interventions implemented in many FBOs to address cardiovascular risk factors have proven successful, including smartphone-based guidance (52). Among the successful smartphone-based interventions is Fostering African American Improvement in Total Health. (FAITH!) The hypertension app, in conjunction with wireless blood pressure monitoring of SDOH, has demonstrated a feasible means of surveillance for CHW-led interventions, which may be necessary for future studies (52). Organizations should collaborate with faith leaders to integrate health promotion activities into religious programs, facilitate support groups within faith communities for patients with CVD, and utilize faith-based communication channels to disseminate health information.

Generally, impactful CHW initiatives ensure that CHWs stay closely tethered to their communities. Programs that focus on medical practices and procedures rather than the unique characteristics of CHWs, perceive CHWs as merely “extra hands” supporting healthcare providers, or limit CHW engagement with patients and community members, do not gain the trust or support of the community, and eventually fail or worse yet, further damage the community's relationship with the healthcare system While specific models highlight certain aspects of patient engagement, all CHW programs must acknowledge that all CHWs will serve as coaches, educators, and advocates of varying abilities (12, 27, 43–48). Successful programs recognize that each community has unique needs. CHWs address these needs, so there is no “one-size-fits-all” approach to developing, maintaining, and replicating the program's initiatives. They also understand that health interventions are difficult to implement when a community's basic needs are ignored (9).

### Policy implications and recommendations

These recommendations are explicitly U.S.-specific. They operationalize globally proven CHW practices through American payment (Medicare CHI/PIN; Medicaid SPAs, 1115 waivers, MCO contracts), credentialing, and data requirements (see Table 1). Despite CMS expanding CHW reimbursement, community-based programs not affiliated with health systems continue to face persistent funding challenges and insufficient resources for program design and evaluation, among other factors. Federal and local initiatives must focus on establishing sustainable financial solutions rather than relying on temporary grant funding (22, 36, 39, 40). Legislation that standardizes credentialing and certification while preserving the fundamental aspects of CHWs, encourages workforce development initiatives, and strengthens Medicaid coverage should be prioritized (3, 42). Mechanism-specific operational examples appear in Supplementary Table S3.

**Figure 3:**
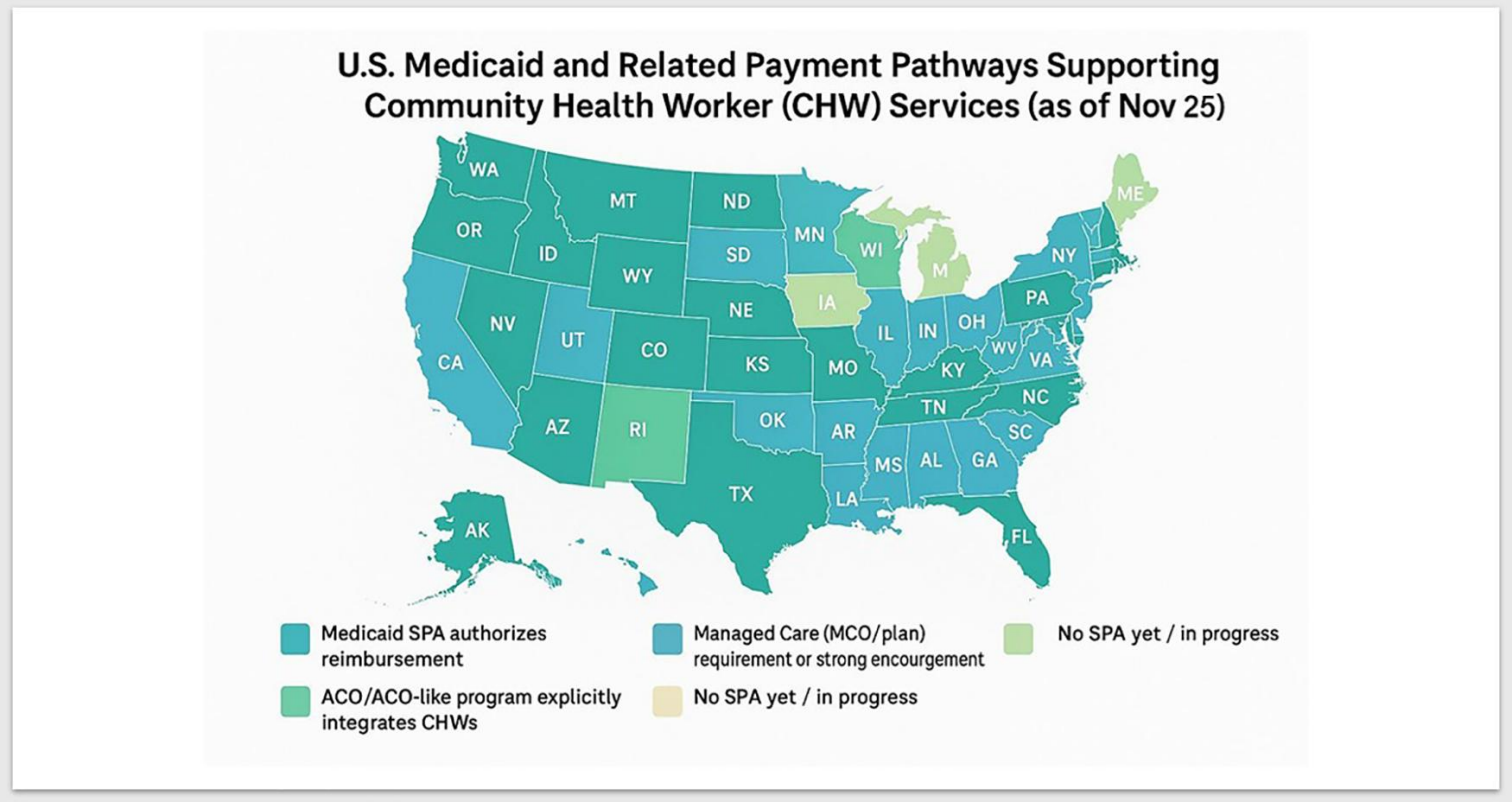
U.S medicaid and managed care organizations encouraged or required to Pay for CHW services by state (*November 2025*). (57).

State Medicaid programs, although encouraged to reimburse comprehensive CHW interventions, have varying success rates (27, 40, 55). Medicare beneficiaries should have access to CHI programs and collaborate with Medicaid-managed care organizations to mitigate financial deficits, enhance coverage, facilitate care coordination, and increase patient engagement. (Figure 3) To address concerns about attrition, providing full-time employment opportunities with benefits, professional development, technical skills training, and supportive administrative and coaching services is essential. Collaboration with the National Association of Community Health Workers (NACHW) will ensure that CHWs play a vital role in the development, implementation, integration, sustainability, and management of CHW programs. CHWs must adapt to mixed environments where they work alongside other healthcare practitioners who may not fully understand the unique needs of CHWs (6, 9, 30, 42, 43).

CHWs, like other frontline public health workers, should not be overlooked in policy, legislation, and regulatory discussions. Instead, they should play prominent roles in conversations about capacity-building, structural sustainability, and implementation. Initiatives specifically focused on increasing access to community-based services, preventive care, obstetric care, and chronic disease management are more likely to succeed when strategically integrated by CHWs (7, 42, 45, 56). However, further research is needed to assess the cost-effectiveness of reimbursement models for CHW services (26). Comparative studies that assess value-based care vs. fee-for-service models could provide additional insight into the sustainability of CHW programs.

### Future directions for practice and research

There is a vast array of research opportunities and continued clinical program development for CHW-led interventions to reduce adverse CVD outcomes (25, 27, 30, 50) (see Table 2). Collaborative partnerships, including various sectors such as Medicaid health plans, local health departments, CBOs, and academic institutions, are crucial in improving healthcare outcomes. By implementing and evaluating CHW-led health plan programs, these collaborations can be used to study the long-term impact on patient-centered outcomes. However, despite improved outcomes observed in local CHW-led interventions, evidence of long-term, longitudinal effects and sustained improvements remains limited. Future research efforts should explore the benefits of a standardized cardiac care-trained CHW. Through this coordinated effort, valuable evidence can be generated to inform effective resource allocation. Furthermore, these partnerships will engage CHWs as integral research partners, ensuring that their insights and experiences contribute to the evaluation process and promote a more inclusive approach to healthcare.

**Table 2:** Future directions for CHW practice and research.

Domain	Immediate actions (≤12 months)	Long-term actions (≥2 years)	Example metrics to track
Recruitment’s training	Inclusive workforce pipelines with shadowing/internships Highlight CHW skillsets in hiring criteria Community-college/on-ramp certificates Hybrid/on-demand training modules	Disease-specific cardiac curriculum (e.g., SMBP onboarding, adherence coaching, transitions) Laddered CHW roles and supervision model Alignment with C3 competencies and state credentials	12-mo CHW retention (%) Trainees completing competency checklists (%) Time-to-hire (days) CHW satisfaction score
Program development	Stand up telehealth-enabled CHW services Individualized care-management workflows Capture CHW best practices/playbooks	Holistic, person-centered programming (CBO/FBO delivery where appropriate) Digital CHW platforms (data capture, prompts)	Enrollment reach (eligible pts engaged, %) Program fidelity score Patient activation/knowledge scores
Sustainability	Activate Medicaid coverage (SPA/MCO) where available Secure cross-sector partnerships (health plans, public health, CBOs) Start adaptive monitoring C evaluation	Personalized CHW support (mentoring, supervision) Staff wellness/burnout prevention Multi-payer funding blend	Reimbursed CHW encounters (% of total) Cost per patient with BP controlled ROI or cost-effectiveness signal CHW turnover rate
Organizational readiness	Seat CHWs on committees/leadership forums Integrate CHW content into manager training Cross-discipline knowledge sharing Set realistic program expectations	Pilot innovative CHW practice models (clinic-embedded, payer- based, public-health, CBO/FBO) with spread plans	Leadership positions held by CHWs (n) Time from referral → first CHW contact (days) Team climate/collaboration scores
Research opportunities	Faith-based and community-based CHW interventions in CVD Digital health CHW pilots Early cost analyses vs. clinician-led models	Pragmatic trials and implementation studies of CHW- led CVD programs CHWs as research partners (co-PIs, co- authors) Comparative cost-effectiveness of task bundles	Studies launched/completed (n) Effect sizes (BP control, adherence) Cost-effectiveness/ROI reported CHW authorship participation (yes/no)

CHW, community health worker; C3, CHW core consensus project; CBO, community-based organization; FBO, faith- based organization; SPA, medicaid state plan amendment; MCO, managed care organization; SMBP, self-measured blood pressure; ROI, return on investment. Metrics are examples—select those aligned with your health-system and payer contracts.

### Persistent evidence gaps

Few studies report long-term clinical and cost outcomes of specific CHW task bundles for CVD; standardized cardiac training modules and supervision models are variably described; and economic evidence remains sparse relative to implementation studies. Priorities include pragmatic trials and comparative cost-effectiveness analyses of clinic-embedded, community-based, and payer-based models, with equity-stratified outcomes. U.S. translation: each transferable global practice should be mapped to the U.S. scope-of-practice, supervision, documentation, and payment (27, 29, 55).

## Conclusion

Integrating CHWs into the chronic management of CVD can strengthen equitable measures that align with standardized cardiac training, are embedded within coordinated care teams, and are supported by sustainable reimbursement. Enhanced, integrated programs with CHWs have demonstrated improvements in cardiovascular outcomes. Recruiting CHWs within a community guarantees cultural competency and trust. This should be conducted with local health agencies, non-profit organizations, and faith-based entities. U.S. implementation requires alignment with adult learning principles, structured supervision, and community partnership models, including faith-based organizations, to ensure cultural relevance and trust.
