# Supplementary material for: Bridging communities, prevention, and heart health: U.S. strategies for CHW cardiovascular training and integration
Source: Front Epidemiol. 2025 Dec 16;5:1597970. doi: 10.3389/fepid.2025.1597970 (PMC12748223; doi:10.3389/fepid.2025.1597970)
Supplement: Supplementary file 1 [file Datasheet1.pdf]

Supplemental Table 3. U.S. Medicaid C-related payment pathways map

Financing Mechanism	State Adoption Status	Operational Requirements & Policy Examples
**Medicaid State Plan Amendment (SPA)**	**MEDICAID REIMBURSED SERVICES:**<br>CA, CO, GA, KS, KY, LA, ME, MI, MN, NM, NV, NY, OK, OR, RI, SD, WA	**Authorization:** Establishes CHW services as a distinct, reimbursable statewide benefit (fee-for-service or capitated).<br>**Provider Qualifications:** States typically require certification or specific training (e.g., C3 alignment) to bill.
**Managed Care (MCO) Requirements**	**MANDATED/ENCOURAGED:**<br>CA, KS, LA, MI, NV, OK, WA	**California:** Plans must create billing pathways and provide education for the CHW benefit.<br>**Michigan:** Plans must *employ* CHWs at defined ratios (e.g., 1 CHW per 5,000 members).<br>**Nevada:** Managed Care Entities (MCEs) must cover CHWs as a recognized provider type.<br>**Washington/Kansas:** MCOs authorized to reimburse CHWs using administrative or quality-improvement funds.<br>**Oklahoma:** Plan-specific pilots (e.g., CHW home visits).
**ACO s Value-Based Models**	**INTEGRATED MODELS:**<br>CO, OR, RI	**Oregon:** Coordinated Care Organizations (CCOs) must include Traditional Health Workers on care teams.<br>**Rhode Island:** Medicaid Accountable Entities (AEs) utilize transformation funds to support CHW integration.<br>**Colorado:** Regional Accountable Entities (RAEs) fund navigation and coordination alongside the SPA.

**Emerging / Alternative Pathways**	**IN DEVELOPMENT/NO SPA**<br><br>ND	**North Dakota:** Reimbursement available for Community Health Representative (CHR) targeted case management; complete CHW SPA under consideration.
**No statewide CHW Program**	Alabama; West Virginia; Wyoming	CDC 2023 CVD Disease Burden: Alabama: Q4 (highest quartile).<br><br>West Virginia: Q4 (highest quartile). Wyoming: Q3 (above-median).
**All other states/DC**	NASHP lists statewide CHW association/coalition	Mixed CVD burden; ranges from Q1 (lowest) to Q4 (highest) across states.
